# Tenecteplase in acute ischemic stroke: a new era in thrombolysis

**DOI:** 10.1055/s-0045-1808088

**Published:** 2025-06-01

**Authors:** Gisele Sampaio Silva, Eva Rocha, Octávio Marques Pontes-Neto, Sheila Ouriques Martins

**Affiliations:** 1 Universidade Federal de São Paulo, Escola Paulista de Medicina, Departamento de Neurologia e Neurocirurgia, São Paulo SP, Brazil.; 2 Hospital Israelita Albert Einstein, Centro de Ensino e Pesquisa, São Paulo SP, Brazil.; 3 Universidade de São Paulo, Faculdade de Medicina de Ribeirão Preto, Departamento de Neurociências e Ciências do Comportamento, Ribeirão Preto SP, Brazil.; 4 Universidade Federal do Rio Grande do Sul, Hospital de Clínicas de Porto Alegre, Departamento de Neurologia, Porto Alegre RS, Brazil.

**Keywords:** Ischemic Stroke, Thrombolytic Therapy, Tenecteplase

## Abstract

Tenecteplase (TNK) is a genetically engineered variant of alteplase, showing promise for acute ischemic stroke treatment. With a longer half-life and higher fibrin specificity, TNK enables more targeted and efficient clot dissolution. Clinical trials demonstrate potential advantages, including improved reperfusion rates and functional outcomes with lower systemic bleeding. Though not officially approved for this purpose by all regulatory agencies, TNK is used off-label and in acute stroke guidelines due to its ease of administration and effectiveness. The 0.25 mg/kg dosage within 4.5 hours of symptom onset was shown to be consistently effective and safe. Further trials are expected to identify patient subgroups that benefit most from TNK treatment. The present narrative review assesses the existing literature and evidence regarding the use of tenecteplase for the treatment of acute ischemic stroke.

## INTRODUCTION

The therapeutic landscape for acute ischemic stroke has evolved considerably, mainly due to advancements in both pharmacologic and mechanical interventions. Intravenous thrombolysis with tissue plasminogen activator (tPA) remains the gold standard for eligible patients within 4.5 hours of symptom onset. 1 2 The extension of this window in certain cases, supported by imaging biomarkers indicating viable brain tissue, has broadened treatment eligibility. 3 4 Mechanical thrombectomy up to 24 hours from symptom onset has redefined outcomes for a significant subset of stroke patients. 5 6 Recent trials have continued to refine the criteria for patient selection, emphasizing the importance of comprehensive vascular imaging and swift workflow protocols. 7 8 Management of acute stroke demands a rapid, coordinated approach that maximizes the use of existing therapeutic options while continuously integrating emerging evidence into practice. 9

Tenecteplase (TNK), a genetically engineered variant of tPA, is emerging as a promising therapeutic option in the treatment of acute ischemic stroke. Developed to have a longer half-life and greater fibrin specificity than alteplase, TNK facilitates a more targeted and efficient dissolution of clots. 10 11 Clinical trials have demonstrated the potential advantages of TNK over tPA, including improved reperfusion rates and functional outcomes with lower rates of systemic bleeding. 10 12 13 14 15 16 17 18 This is a narrative review of the current literature with updates on the treatment of acute ischemic stroke with TNK.

## CHALLENGES IN ACUTE ISCHEMIC STROKE TREATMENT

The treatment of acute ischemic stroke presents several challenges that impact patient outcomes significantly. One of the primary difficulties is the narrow time window available for effective intervention. 9 This time sensitivity requires rapid diagnosis and decision-making, which can be hindered by delayed patient presentation to the hospital and the time it takes to perform necessary imaging studies. 19

Additionally, there is the logistical challenge of providing rapid, coordinated care. This involves the immediate healthcare team, emergency medical services, and the broader hospital infrastructure, which must facilitate urgent imaging and treatment. Moreover, disparities in access to stroke care, particularly in rural or underserved regions, exacerbate these challenges and can lead to significant differences in outcomes among populations. 19 20

Addressing these challenges requires continuous improvements in stroke care protocols, training for healthcare providers, public education to increase awareness of stroke symptoms, and enhanced systems for rapid patient transport and treatment. These improvements are crucial for increasing the effectiveness of stroke treatments and improving survival and recovery rates. 19 20 21

The use of TNK could help address several challenges in the treatment of acute ischemic stroke. First, its administration involves a single bolus dose, which simplifies and speeds up the treatment process compared with the infusion required for alteplase. This can significantly reduce the door-to-needle time, a critical factor in stroke management, during which every minute counts. Additionally, TNK-enhanced fibrin specificity and longer half-life may improve reperfusion rates, potentially leading to better outcomes for patients with large vessel occlusions, which are critical candidates for rapid reperfusion therapy. 11 22

## TENECTEPLASE: AN OVERVIEW

Endogenous tPA is a serine protease produced by endothelial cells. It plays a crucial role in coagulation homeostasis by converting plasminogen into plasmin, degrading fibrin within thrombi. 23 The development of recombinant DNA technology has facilitated the production of this wild-type tPA, making it possible to use therapeutic fibrinolysis to target arterial thrombi and treat acute ischemic conditions effectively. Tenecteplase, a variant of tissue plasminogen activator, is engineered with three amino acid modifications relative to alteplase. 11 These structural modifications improve its pharmacodynamic and pharmacokinetic profiles, offering enhanced therapeutic benefits. 24

Tenecteplase features an extended half-life and reduced plasma clearance rate, allowing for its administration in a single bolus rather than the continuous infusion required by alteplase, which is particularly advantageous during interhospital transfers ( Table 1 ). 22 Additionally, TNK demonstrates a more than 15-fold increase in fibrin specificity and an 80-fold greater resistance to plasminogen activator inhibitor-1 (PAI-1) than alteplase. 25 The pharmacodynamic and pharmacokinetic advantages of TNK over alteplase have been substantiated through animal studies. Tenecteplase exhibits a significantly slower clearance rate in rabbits, highlighting its sustained activity, as opposed to the faster clearance observed with alteplase. Tenecteplase also has enhanced fibrin specificity, affecting fibrinogen, plasminogen, and α2-antiplasmin levels to a lesser extent than alteplase, suggesting a more targeted action. Additionally, unlike alteplase, TNK does not promote platelet aggregation facilitated by collagen or arachidonic acid at the thrombolysis site, which reduces the likelihood of reocclusion in recanalized vessels. This profile suggests that TNK could be more effective in managing platelet-rich clots without compromising safety. 11 25

**Table 1:** Comparison between tenecteplase and alteplase in the treatment of acute ischemic stroke

Medication	Alteplase	Tenecteplase
**Synthesis**	Recombinant version of tPA	Bioengineered version of alteplase
**FDA approved indications**	Acute ischemic stroke Myocardial infarction Acute pulmonary embolism	Myocardial infarction
**EMA approved indications**	Acute ischemic stroke Myocardial infarction Acute pulmonary embolism	Acute ischemic stroke Myocardial infarction
**Administration**	10% bolus followed by 1-h infusion	Single bolus
**Fibrin specificity**	Lower than Tenecteplase	14–15x higher than alteplase
**Degradation**	Less resistant to PAI-1	80x higher resistance to PAI-1
**Plasma half-life**	4–6 minute	17–20 min

Abbreviations: EMA, European Medicines Agency; FDA, Food and Drug Administration; PAI-1, plasminogen activator inhibitor-1; tPA, tissue plasminogen activator.

Tenecteplase, known commercially as TNKase in the United States by Genentech, and Metalyse in Europe, by Boehringer Ingelheim, received regulatory approval in 2000 for ST-segment elevation acute myocardial infarction (STEMI). This medication is prescribed in a tiered, weight-based dosage—starting at 0.5 mg/kg and capping at 50 mg—and is administered as a rapid 5 to 10-second bolus specifically for treating STEMI. 26 As of January 2024, the European Medicines Agency has approved the use of TNK for stroke treatment. 27 In India, TNK is available under various brand names for treating STEMI and stroke, although the dosages vary. 26 Boehringer Ingelheim's in vitro studies suggest that the Indian version might be less pure and less effective in thrombolysis, which has sparked debates on its status as a biosimilar. 28 Additionally, TNK is also produced in China. The Chinese medication was recently evaluated in extensive phase-II and -III stroke clinical trials. 29

## TENECTEPLASE IN STROKE CLINICAL TRIALS

Recently, many clinical trials have evaluated TNK in acute ischemic stroke ( Table 2 ). Phase two trials tested different doses of TNK and assessed safety, efficacy, and reperfusion rates. In 2010, the Study of Tenecteplase in Acute Ischemic Stroke (TNK-S2B) trial tested 3 doses of TNK (0.1, 0.25, and 0.4 mg/kg) against alteplase and showed that TNK was safe and similarly effective. 12 Two years later, the Tenecteplase Versus Alteplase for Acute Ischemic Stroke (TAAIS) trial showed greater reperfusion rates in a pooled group of TNK with 0.1 and 0.25 mg/kg doses compared with alteplase. 10 The TNK-Tissue-Type Plasminogen Activator Evaluation for Minor Ischemic Stroke With Proven Occlusion (TEMPO-1) was a small phase-2 trial evaluating the safety and feasibility of TNK in patients with minor stroke arriving within 12 hours of symptom onset and showed good recanalization rates in both dosages. 13 The Alteplase-Tenecteplase Trial Evaluation for Stroke Thrombolysis (ATTEST) trial evaluated penumbra salvage with a 0.25 mg/kg dosage, and the Tenecteplase versus alteplase for the management of acute ischemic stroke in Norway ( NORTEST) trial evaluated a 0.4 mg/kg dosagefor improving functional outcomes, compared with 0.9 mg/kg of alteplase. 14 15 The studies did not demonstrate that TNK was superior to alteplase but indicated similar safety outcomes.

**Table 2:** Clinical trials evaluating tenecteplase in acute ischemic stroke

Trial name/Phase	Year published	Number of patients	Intervention	Inclusion criteria	Results	Safety outcomes
TNK-S2B 12 Phase IIB/III	2010	112	TNK: 0.1 mg/kg 0.25 mg/kg 0.4 mg/kg r-tPA: 0.9 mg/kg	Time window: 0–3 hours. NIHSS: Aphasia score > 1, motor power > 1, vision > 2, or attention > 2.	No differences in 3-month functional outcome (mRS score ≥ 4) between remaining TNK doses and alteplase.	Symptomatic ICH: TNK 0.4 mg/kg: 3/19 (15.8%) TNK 0.25 mg/kg: 2/31 (6.5%) TNK 0.1 mg/kg: 0/31 (0%) r-tPA: 1/31 (3.2%)
TAAIS trial 10 Phase IIB	2012	75	TNK: 0.1 mg/kg 0.25 mg/kg r-tPA: 0.9 mg/kg	Time window 0–6 hours. NIHSS > 4 Maximum age: 85. Visible occlusion on CTA > 20% mismatch on CTP. Prestroke mRS: ≤ 2.	Pooled TNK groups (0.1 and 0.25 mg/kg) had greater reperfusion and clinical improvement at 24 hours compared with the alteplase group (79.3 ± 28.8% vs 55.4 ± 38.7%, *p* = 0.004 and (8.0 ± 5.5% vs 3.0 ± 6.3%, *p* < 0.001).	Symptomatic ICH: 3/25 (12%) alteplase and 2/50 (4%) TNK pooled. No differences in mortality were found at 90 days.
TEMPO-1 13 Phase II	2015	50	TNK: 0.1 mg/kg 0.25 mg/kg	Time window: 0–12 hours. NIHSS ≤ 5. Visible occlusion on CTA.	66% had mRS 0–1 at 90 days. Recanalization rates were high in 0.1 mg/kg (39% complete and 17% partial) and 0.25 mg/kg (52% complete and 9% partial). Complete recanalization was significantly related to mRS 0–1 at 90 days ( *p* = 0.026)	In 0.25 mg/kg group there was 1 symptomatic ICH (4%)
ATTEST 14 Phase II	2015	104	TNK: 0.25 mg/kg r-tPA: 0.9 mg/kg	Time window: 0–4.5 hours. NIHSS: ≥ 1. Perfusion imaging: > 20% mismatch.	No significant differences for percentage of penumbra salvaged between TNK 68% (28/47) and alteplase 68% (23/49) groups ( *p* = 0.81)	No differences were found in mortality and rates of symptomatic ICH
NOR-TEST 15 Phase III	2017	1,050	TNK: 0.4 mg/kg r-tPA: 0.9 mg/kg	Time window 0–4.5 hours. NIHSS ≥ 1. Wake-up patients: DWI/FLAIR mismatch required. Prestroke mRS ≤ 2.	Superiority trial. Good functional outcome (mRS score ≤ 1) at 90 days achieved by 354/549 (64%) of tenectplase and 345/551 (63%) of alteplase groups, *p* = 0.52, with OR, 1.8 [0.84–1.38]	No difference in symptomatic ICH and overall mortality In moderate to severe strokes mortality increased in the TNK group (26.3% vs 9.1%; *p* = 0.045) at 90 days
EXTEND-IA TNK 16 Phase II	2018	202	TNK: 0.25 mg/kg r-tPA: 0.9 mg/kg	Time window: 0–4.5 hours thrombolysis, EVT ≤ 6 hours. NIHSS: ≥ 1 ICA, M1, M2, or basilar artery occlusion on CTA or MRA Prestroke mRS ≤ 3.	The primary outcome (mTICI 2b or 3) occurred in 22/101 (22%) of patients treated with TNK vs 10/101 (10%) of those treated with r-tPA ( *p* = 0.002 for non-inferiority; *p* = 0.03 for superiority)	No difference in symptomatic ICH or mortality
EXTEND-IA TNK part 2 30 Phase II	2020	300	TNK: 0.25 mg/kg 0.40 mg/kg	Time window: 0–4.5 hours. ICA, M1, M2, or basilar artery occlusion on CTA or MRA. TNK given before mechanical thrombectomy.	Reperfusion greater than 50% of the previously occluded vascular territory was 29 of 150 (19.3%) in the 0.40 mg/kg group vs 29 of 150 (19.3%) in the 0.25 mg/kg group. There were no significant differences in any of the 4 functional outcomes between the 0.40 mg/kg and 0.25 mg/kg groups.	There were no significant differences in all-cause deaths (26 [17%] vs 22 [15%]; unadjusted risk difference, 2.7% [95% CI, -5.6– 11.0%]) or symptomatic ICH (7 [4.7%] vs 2 [1.3%]; unadjusted risk difference, 3.3% [95% CI, -0.5– 7.2%]).
AcT 33 Phase IIB/III	2022	1,600	TNK: 0.25 mg/kg r-tPA: 0.9 mg/kg	Time window: 0–4.5 hours.	mRS 0–1 at 90–120 days: 36.9% of patients in the TNK group and 34.8% patients in the alteplase group (meeting the prespecified non-inferiority threshold).	3.4% of patients in the TNK group and 3.2% of patients in the alteplase group had 24 h symptomatic ICH 15.3% in the TNK group and 15.4% in the alteplase group died within 90 days of starting treatment.
NOR-TEST 2A 32 Phase III	2022	216 (stopped after safety review)	TNK: 0.4 mg/kg r-tPA: 0.9 mg/kg	Time window: 0–4.5 hours. NIHSS ≥ 6. Wake-up patients: DWI/FLAIR mismatch required. Prestroke mRS ≤ 2.	mRS score of 0–1 at 90 days: 32% TNK vs 51% alteplase group ( *p* = 0.006).	Any ICH: 21% in the TNK group and 7% in the alteplase group ( *p* = 0.0031). Mortality at 3 months: 16% in the TNK group and 5% in the alteplase group ( *p* = 0.013). Symptomatic ICH: 6% in the TNK group and 1% in the alteplase group ( *p* = 0.061)
TRACE 48 Phase II	2022	240	TNK: 0.1 mg/kg 0.25 mg/kg 0.32 mg/kg r-tPA: 0.9 mg/kg	Time window: 0–3 hours.	There was no difference in the improvement on National Institutes of Health Stroke Scale on day 14 in the 3 tiers and control group (63.3%, 77.2%, 66.7% vs 62.7%).	The number of sICH was 3 of 60 (5.0%) in the 0.1 mg/kg group, none in the 0.25 mg/kg group, 2 of 60 (3.3%) in the 0.32 mg/kg group and 1 (1.7%) of 59 in the r-tPA group. There were no significant between-group differences in severe adverse events
TASTE A 17 Phase II	2022	104	TNK: 0.25 mg/kg r-tPA: 0.9 mg/kg	Time window: 0–4.5 hours.	On arrival at the hospital, the perfusion lesion volume was significantly smaller with tenecteplase (median 12 mL [IQR 3–28]) than with alteplase (35 mL [18–76]; adjusted incidence rate ratio 0.55, 95% CI 0.37–0.81; *p* = 0.0030). At 90 days, an mRS of 5 or 6 was reported in eight (15%) patients allocated to tenecteplase and ten (20%) patients allocated to alteplase (adjusted odds ratio [aOR] 0.70, 95% CI 0.23–2.16; *p* = 0.54)	Five (9%) patients allocated to tenecteplase, and five (10%) patients allocated to alteplase died from any cause at 90 days (aOR 1.12, 95% CI 0.26–4.90; *p* = 0.88). No cases of symptomatic intracerebral hemorrhage were reported within 36 hours with either treatment.
TRACE-2 ^18^ Phase III	2023	1,430	TNK: 0.25 mg/kg r-tPA: 0.9 mg/kg	Time window: 0–4.5 hours. Prestroke mRS ≤ 1 NIHSS 5–25. Ineligible for mechanical thrombectomy.	mRS score of 0–1 at 90 days: 62% of the TNK group and 58% of the alteplase group (greater than the non-inferiority margin)	No difference in symptomatic ICH within 36 hours: 2% in the TNK group and 2% in the alteplase group. No difference in mortality within 90 days: 7% in the TNK group versus 5% in the alteplase group.
TWIST 43 Phase III	2023	578	TNK: 0.25 mg/kg control: no thrombolysis	Time window: wake-up strokes with limb weakness within 4.5 h of awakening. NIHSS ≥ 3 or aphasia. Non-contrast CT.	Treatment with tenecteplase was not associated with better functional outcome, according to mRS score at 90 days (adjusted OR 1.18, 95% CI 0.88–1.58; *p* = 0.27).	Mortality at 90 days did not significantly differ between groups: 10% in the TNK group and 8% in the control group ( *p* = 0.37). Symptomatic ICH occurred in 2% of patients in the TNK group versus 1% in the control group ( *p* = 0.28).
BRETIS-TNK Phase IIB	2023	26	AIS-LVO patients with large-artery atherosclerosis etiology	Intra-arterial TNK (4 mg) after microcatheter navigation through the clot was administered, followed by TNK (0.4 mg/min) given continuously for 20 minutes after the first retrieval attempt of EVT. Comparison: historical cohort from 2015 to 2019.	The first-pass successful reperfusion rate was higher in the TNK versus control group (53.8% versus 36%, *p* = 0.14), and the difference became statistically significant after propensity score matching (53.8% versus 23.1%, *p* = 0.03). There was a trend toward higher proportion of functional independence at 90 days in the TNK compared with the control group (50% versus 32%, *p* = 0.11)	There was no difference in symptomatic ICH between the TNK and control groups (7.7% versus 10.0%, *p* = 0.92).
ATTEST-2 Phase III	2023	1,858	TNK: 0.25 mg/kg r-tPA: 0.9 mg/kg	Time window: 4.5 hours.	90-day mRS score distribution: acOR 1.07 (95%CI 0.90–1.27); Non-inferiority *p* < 0.0001; Superiority *p* = 0.456	sICH (ECASS-3): 29/885 (3.3%) versus 21/891 (2.4%), OR 1.44 (95%CI 0.81–2.56), *p* = 0.212 mRS 5–6: 95/885 (10.7%) versus 111/891 (12.5%). Death: 68/885 (7.7%) versus 75/891 (8.4%), OR 0.96 (95%CI 0.69–1.33), *p* = 0.797
TIMELESS 40 Phase III	2024	458	TNK: 0.25 mg/kg Placebo	Time window: 4.5–24 hours. Prestroke mRS: ≤ 1. NIHSS: 5–25. MCA or ICA occlusion.	The adjusted common odds ratio for the distribution of scores on the mRS at 90 days for TNK as compared with placebo was 1.13 (95% confidence interval, 0.82 to 1.57; *p* = 0.45).	In the safety population, mortality at 90 days was 19.7% in the TNK group and 18.2% in the placebo group, and the incidence of symptomatic ICH was 3.2% and 2.3%, respectively.
TASTE 34 Phase III	2024	680	TNK: 0.25 mg/kg r-tPA: 0.9 mg/kg	Time window: 0–4.5 hours. Core on DWI/CT *p* < 70 mL. Penumbra mismatch. Ratio > 1.8 and > 15 mL volume.	Primary outcome: mRS 0–1 at 3 months (non-inferiority) - Intention to treat population: 57% TNK and 55% alteplase (OR 1.19; *p* = 0.03) - Per protocol population: 59% in TNK and 56% in rtPA (OR 1.27, *p* = 0.01)	
TRACE-III 41 Phase III	2024	506	TNK: 0.25 mg/kg Placebo	Time window: 4.5–24 hours. Prestroke mRS score < 1. Baseline NIHSS 6–25. Neuroimaging: ICA, MCA M1 or M2 occlusion confirmed by CTA/MRA, ICA, MCA M1 or M2 being responsible for signs and symptoms of acute stroke. Target mismatch profile on CTP or MRl + MR perfusion (ischemic core volume < 70 ml, mismatch ratio > 1.8, and mismatch volume > 15 ml).	Primary outcome: mRS 0–1 at 3 months: 33% in TNK group and 24% in rtPA group ( *p* = 0.03)	Symptomatic ICH 3% vs 0.8% No difference in mortality.
TEMPO-2 *36 Phase III	2024	886	TNK: 0.25 mg/kg Placebo	Time window: 0–12 hours. Prestroke mRS 0–2. NIHSS 0–5. lntracranial occlusion or focal perfusion abnormality ASPECTS > 6.	Primary outcome: return to baseline function (75% control and 72% TNK)	
CHABLIS-T 29 Phase III	2024	86	TNK: 0.25 mg/kg 0.32 mg/kg	Time window: 4.5–24 hours. NIHSS > 5 CTA: IAO ICA, MCA M1/M2, ACA. Perfusion mismatch > 1.2, core < 70, penumbra > 10.	Primary outcome(major reperfusion without the occurrence of symptomatic ICH): 14 (32.6%) in the 0.25 mg/kg and 10 (23.3%) in the 0.32 mg/kg dosage. mRS 0–1 for 90 days. 12 (27.9%) in the 0.25 mg/kg and 21 (48.8%) in the 0.32 mg/kg dosage.	Similar rates of symptomatic ICH (4.3% vs 4.3%).
CHABLIS T II *42 Phase III	2024	224	TNK: 0.25 mg/kg Best medical treatment (including alteplase and thrombectomy when eligible)	Time window: 4.5–24 hours. Anterior large or medium vessel occlusion. Ischemic core volume < 70 mL, and penumbral mismatch > 10 mL measured on CTP.	The primary outcome was major reperfusion without symptomatic ICH at 24–48 hours. It was achieved in 33% of patients in the TNK group and 10% in best medical treatment group, with an adjusted risk ratio of 3.0 (95% CI, 1.5–5.7; *p* = 0.001). There were no significant differences between groups in secondary outcomes including NIHSS score at 24–48 hours, infarct growth at 3–5 days, and mRS score at 90 days.	There was no significant difference between groups regarding sICH at 24–48 hours, any intracranial hemorrhage, parenchymal hematoma type 2, and 90-day mRS 5–6.
ORIGINAL *35 Phase III	2024	1,489	TNK: 0.25 mg/kg r-tPA: 0.9 mg/kg	Time window: 0–4.5 hours.	The primary outcome was the proportion of patients with an mRS score of 0 to 1 at 90 days, with 72.7% of TNK-treated patients and 70.3% of alteplase-treated patients meeting that standard (adjusted risk ratio 1.02; 95% CI 0.96–1.09).	The rate of symptomatic intracranial hemorrhage was not significantly different between groups, irrespective of the definition use. Using ECASS III criteria, for example, the rate was 1.2% in both arms. There also were no differences between the TNK and alteplase arms in the rate of all-cause mortality at 90 days (4.6% vs 5.8%) or serious adverse events (15.8% vs 15.6%).

Abbreviations: ACT, intravenous tenecteplase compared with alteplase for acute ischemic stroke in Canada; AIS, acute ischemic stroke; aOR, adjusted odds ratio; ASPECTS, Alberta stroke program early CT score; ATTEST, Alteplase-Tenecteplase Trial Evaluation for Stroke Thrombolysis; BRETIS-TNK, Boosting REcanalization of Thrombectomy for Ischemic Stroke by Intra-arterial TNK; CHABLIS T II trial, CHinese Acute Tissue-Based Imaging Selection for Lysis In Stroke Tenecteplase II; CTA, computed tomography angiography; CTP, computed tomography perfusion; DWI, diffusion-weighted imaging; ECASS III, European Cooperative Acute Stroke Study III; EXTEND-IA TNK, Tenecteplase versus Alteplase before Endovascular Therapy for Ischemic Stroke; EVT, endovascular thrombectomy; FLAIR, fluid-attenuated inversion recovery; ICA, internal carotid artery; ICH, intracranial hemorrhage; LVO, large vessel occlusion; MCA, middle cerebral artery; MRI, magnetic resonance imaging; mRS, modified Rankin Scale; mTICI, modified Treatment in Cerebral Ischemia; NIHSS, National Institutes of Health Stroke Scale; NOR-TEST, Norwegian Tenecteplase Stroke Trial; OR, odds ratio; ORIGINAL, Tenecteplase versus alteplase in Chinese patients with acute ischemic stroke; rtPA, recombinant tissue plasminogen activator; TAAIS, Tenecteplase Versus Alteplase for Acute Ischemic Stroke; and TNK-S2, Study of Tenecteplase [TNK] in Acute Ischemic Stroke; TRACE, Tenecteplase Reperfusion Therapy in Acute Ischemic Cerebrovascular Events; TWIST, Safety and efficacy of tenecteplase in patients with wake-up stroke assessed by non-contrast CT; TIMELESS Tenecteplase for Stroke at 4.5 to 24 Hours with Perfusion-Imaging Selection; TEMPO-1, TNK-Tissue-Type Plasminogen Activator Evaluation for Minor Ischemic Stroke With Proven Occlusion; TASTE, The Tenecteplase versus Alteplase for Stroke Thrombolysis Evaluation.

Note: *The results were presented at conferences and are pending publication.

Both TAAIS and Tenecteplase versus Alteplase before Thrombectomy for Ischemic Stroke (EXTEND-IA TNK) trials suggested that patients with large vessel occlusion had improved functional outcomes when treated with TNK compared with alteplase. 10 16 The EXTEND-IA TNK trial determined the inclusion of TNK in the 2019 American Heart Association acute stroke guidelines as an option for treating patients undergoing thrombectomy. 9 This phase-2 non-inferiority trial showed that recanalization occurred in 22/101 (22%) of patients treated with 0.25 mg/kg of TNK vs 10/101 (10%) of those treated with alteplase ( *p* = 0.002 for non-inferiority; *p* = 0.03 for superiority). Functional outcomes were improved with TNK when comparing the median 90-day modified Rankin scale (mRS) for TNK and alteplase (2 vs 3, *p* < 0.04). Additionally, there was lower mortality risk in patients treated with TNK in the EXTEND-IA TNK trial. The EXTEND-IA TNK part 2 trial, published in 2020, evaluated 300 patients within a 4.5-hour time window who had large vessel occlusion and received 0.25 or 0.4 mg/kg of TNK prior to undergoing mechanical thrombectomy. 30 Reperfusion rates and functional outcomes were similar between the two groups. A pooled analysis of EXTEND-IA TNK trials showed higher rates of symptomatic ICH and mortality with the dose of 0.4 mg/kg compared with 0.25 mg/kg of TNK. 31

The Comparison of Tenecteplase with Alteplase for the Early Treatment of Ischemic Stroke in the Melbourne Mobile Stroke Unit (TASTE-A) was a phase-2 trial published in 2022 evaluating reperfusion rates in patients who were treated with TNK compared with alteplase in a mobile stroke unit. It showed improved reperfusion (lesion size 12 mL vs 35 mL, *p* < 0.003) with similar safety outcomes. 17 In the same year, NOR-TEST 2 showed that the higher dosage of TNK (0.4 mg/kg) led to worse functional and safety outcomes than alteplase in moderate to severe strokes. 32 The Intravenous Tenecteplase Compared with Alteplase for Acute Ischemic Stroke in Canada (AcT) trial was a phase-3 non-inferiority trial evaluating 1,600 patients. It showed that TNK was non-inferior to alteplase in routine clinical practice when treating patients within 4.5 h of ischemic stroke (mRS 0–1 at 90–120 days of 36.9% in the TNK group and 34.8% in the alteplase group). 33 The The Tenecteplase versus Alteplase for Stroke Thrombolysis Evaluation (TASTE) and Tenecteplase versus alteplase in Chinese patients with acute ischemic stroke (ORIGINAL) trials were presented in 2024 and showed that TNK was non-inferior to alteplase in the 4.5-hour time window. 34 35 36

In 2023, TRACE-2 and ATTEST-2, 2 phase-3 trials with a total of 3,288 patients, had results presented showing the non-inferiority of TNK 0.25 mg/kg compared with alteplase in acute ischemic stroke within 4.5 hours. 18 37 The TRACE-2 trial showed mRS scores of 0 to 1 in 62% for the TNK group and 58% for the alteplase group (greater than the non-inferiority margin) at 90 days, with no significant difference in safety outcomes. These results provide extraordinary evidence to support the use of TNK at a dose of 0.25 mg/kg within 4.5 hours of acute ischemic stroke as a non-inferior option to alteplase. Consequently, the 2023 European Stroke Organization published a document recommending TNK 0.25 mg/kg as an alternative to 0.9 mg/kg alteplase for patients with acute ischemic stroke within 4.5 hours of symptom onset. 38 The 2023 edition of the National Clinical Guideline for Stroke for the United Kingdom and Ireland also recommended that thrombolysis with alteplase or TNK should be considered for patients with acute ischemic stroke within 4.5 hours of known onset. 39

Although TNK has consistently shown benefits in the 4.5-hour time window, trials evaluating its use in an extended time window have shown conflicting results. The Thrombolysis in Imaging-Eligible, Late-Window Patients to Assess the Efficacy and Safety of Tenecteplase (TIMELESS) trial published in 2024 evaluated TNK in the 4.5 to 24-hour time window in patients with NIHSS of 5 to 25 and large vessel occlusions. The study showed no benefit of TNK in this extended time window. 40 The TRACE-III was a Chinese trial that also evaluated TNK in the extended time window of 4.5 to 24 hours in patients with mismatch ratio > 1.8 and mismatch volume > 15 ml and large vessel occlusion that had not received thrombectomy treatment. The trial showed better functional outcomes in patients receiving TNK compared with placebo (33% vs 24%, *p* = 0.03). 41 The difference between these two trials is that in TIMELESS, most patients (77.3%) received endovascular treatment, which might have influenced the results. This is crucial, particularly in low- and middle-income countries with limited access to endovascular therapies. The CHinese Acute Tissue-Based Imaging Selection for Lysis In Stroke Tenecteplase II (CHABLIS T II) trial showed better recanalization but no improvement in functional outcome between 4.5 and 24 hours. 42 The Tenecteplase in Wake-up Ischemic Stroke Trial (TWIST) trial evaluating TNK within 4.5 h of awakening in patients with wake-up stroke did not show improvement in functional outcomes in 90 days. 43

The effectiveness of tenecteplase in patients with low NIHSS and large vessel occlusion has been studied, but no treatment benefit was demonstrated. The TEMPO-2 trial evaluated 886 patients within 12 hours of acute ischemic stroke with NIHSS 0 to 5 and large vessel occlusion and showed no difference in the primary functionality outcome. 36 The use of intra-arterial TNK has been explored in the BRETIS-TNK trial, which evaluated TNK intra-arterially in patients with large artery atherosclerosis undergoing mechanical thrombectomy and showed a trend to a higher reperfusion rate that was statistically significant after propensity score matching and a trend toward a better functional outcome. 44

Further trials are ongoing evaluating TNK in extended time windows (RESILIENT EXTEND-IV, ETERNAL-LVO, POST-ETERNAL), TNK as bridging therapy to endovascular treatments (DIRECT-TNK, BRIDGE-TNK), and adjunctive intra-arterial TNK (INSIST-IT, INSIST-TNK, ALLY, TECNO, BRETIS-TNK II, RESCUE-TNK, ATTENTION IA, ANGEL-TNK, EXTEND-AGNES TNK). 11

Two recent meta-analyses showed a superiority of TNK over alteplase in ischemic stroke. A 2021 systematic review and meta-analysis of randomized clinical trials evaluating TNK in patients with large vessel occlusions showed a better rate of complete recanalization and neurological improvement after 3 months. 45 Likewise, a 2022 systematic review and meta-analysis of non-randomized studies showed real-world evidence of better recanalization rates and functional outcomes with TNK. 22

## PRACTICAL CONSIDERATIONS FOR THE USE OF TENECTEPLASE

Tenecteplase is easily administered for acute ischemic stroke through a single bolus dosage of 0.25 mg/kg over 5 to 10 seconds with a maximum dosage of 25 mg. It does not require infusion monitoring during transfer, and it may reduce dosing errors and improve patient workflow. The TASTE-A trial showed it can be administered without problems in the prehospital setting. 17 Costs may also be reduced when using TNL as a thrombolytic. Studies have shown that substituting intravenous alteplase with TNK can save approximately $3,000 in the United States. In other countries, switching to TNK can represent a 50% cost reduction. 11

Two ongoing clinical trials in Brazil are investigating the efficacy of administering TNK in different scenarios. The Randomization to Endovascular Treatment Alone or Preceded by Systemic Thrombolysis With Tenecteplase in Acute Ischemic Stroke due to Large Intracranial Vessel Occlusion (RESILIENT DIRECT TNK) trial is assessing the effectiveness of administering TNK prior to mechanical thrombectomy, in patients arriving within 4.5 hours of acute ischemic stroke, compared with placebo. Meanwhile, the RESILIENT EXTEND IV trial focuses on patients who are not undergoing mechanical thrombectomy, examining the effects of TNK administered during a later time window, compared with placebo, specifically between 4.5 and 12 hours after symptom onset.

In the real-world setting, many centers have switched from alteplase to the off-label use of TNK based on clinical trial data and guideline recommendations. 22 However, several countries and institutions still await approval by the corresponding sanitary agency for use in stroke. The European Medicines Agency (EMA) approved TNK for stroke in January 2024, and since then, several European countries have changed their practice to make TNK the first choice for stroke treatment. 27 The only FDA-approved drug for acute stroke treatment is alteplase. Real-world registries support the use of TNK in acute stroke. A multicenter prospective registry of 588 patients with stroke found that TNK had a higher proportion of patients achieving target door-to-needle time within 45 minutes (41% versus 29%, *p* = 0.001) and target door-in-door-out time within 90 minutes (37% versus 14%, *p* = 0.02). Unfavorable outcomes such as symptomatic ICH, in-hospital mortality, or discharge to hospice were numerically lower in the TNK group (7.3% versus 11.9%), and total hospital costs were also lower in the TNK group ($13,382 versus $15,841, *p* < 0.001). 46

Given their similar mechanism, the potential side effects of TNK are similar to those of alteplase. The treatment of post-tenecteplase symptomatic intracranial hemorrhage should also be similar to alteplase-related hemorrhagic transformation, with blood pressure control, cryoprecipitate, and tranexamic acid per the American Heart Association (AHA) guidelines. 47 Healthcare providers need thorough education on the proper use of this new medication to prevent misuse and complications. Future trials are expected to help determine the most suitable patient subgroups for TNK treatment.

In conclusion, TNK has been demonstrated to be a viable, easy-to-administer, and effective drug for managing acute ischemic stroke. Although not officially approved for this purpose by all regulatory agencies, it has been used off-label and even included in acute stroke guidelines. The dosage of 0.25 mg/kg within 4.5 hours of onset of symptoms has consistently shown to be effective and safe. Future trials are expected to further delineate the patient subgroups that would benefit most from TNK treatment.
